# Impaired response of the bronchial epithelium to inflammation characterizes severe equine asthma

**DOI:** 10.1186/s12864-017-4107-6

**Published:** 2017-09-08

**Authors:** Laurence Tessier, Olivier Côté, Mary Ellen Clark, Laurent Viel, Andrés Diaz-Méndez, Simon Anders, Dorothee Bienzle

**Affiliations:** 1 Department of Pathobiology, University of Guelph, 50 Stone Road East, Guelph, ON N1G 2W1 Canada; 2 Department of Clinical Studies, University of Guelph, 50 Stone Road East, Guelph, ON N1G 2W1 Canada; 3 Institute for Molecular Medicine, Finland (FIMM), University of Helsinki, Tukholmankatu 8, 00014 Helsinki, Finland; 4 Present address: BioAssay Works LLC, 10075 Tyler Place, Suite 18, Ijamsville, MD 21754 USA; 5 Present address: Centre for Equine Infectious Disease, The University of Melbourne, Melbourne, VIC 3010 Australia

**Keywords:** Asthma, Bronchus, Differential expression analysis, High-throughput nucleotide sequencing, Horse

## Abstract

**Background:**

Severe equine asthma is a naturally occurring lung inflammatory disease of mature animals characterized by neutrophilic inflammation, bronchoconstriction, mucus hypersecretion and airway remodeling. Exacerbations are triggered by inhalation of dust and microbial components. Affected animals eventually are unable of aerobic performance. In this study transcriptomic differences between asthmatic and non-asthmatic animals in the response of the bronchial epithelium to an inhaled challenge were determined.

**Results:**

Paired endobronchial biopsies were obtained pre- and post-challenge from asthmatic and non-asthmatic animals. The transcriptome, determined by RNA-seq and analyzed with edgeR, contained 111 genes differentially expressed (DE) after challenge between horses with and without asthma, and 81 of these were upregulated. Genes involved in neutrophil migration and activation were in central location in interaction networks, and related gene ontology terms were significantly overrepresented. Relative abundance of specific gene products as determined by immunohistochemistry was correlated with differential gene expression. Gene sets involved in neutrophil chemotaxis, immune and inflammatory response, secretion, blood coagulation and apoptosis were overrepresented among up-regulated genes, while the rhythmic process gene set was overrepresented among down-regulated genes. MMP1, IL8, TLR4 and MMP9 appeared to be the most important proteins in connecting the STRING protein network of DE genes.

**Conclusions:**

Several differentially expressed genes and networks in horses with asthma also contribute to human asthma, highlighting similarities between severe human adult and equine asthma. Neutrophil activation by the bronchial epithelium is suggested as the trigger of the inflammatory cascade in equine asthma, followed by epithelial injury and impaired repair and differentiation. Circadian rhythm dysregulation and the sonic Hedgehog pathway were identified as potential novel contributory factors in equine asthma.

**Electronic supplementary material:**

The online version of this article (10.1186/s12864-017-4107-6) contains supplementary material, which is available to authorized users.

## Background

Severe equine asthma, formerly termed recurrent airway obstruction (RAO) or heaves, is a naturally occurring chronic lung inflammatory disease of horses exposed to airborne molds and particulate material [1]. The condition develops with repeated inhalation of molds and/or dusty air in barns or on pasture in hot and humid climates, as well as in environments with high concentration of fungal spores or grass pollen grains [2]. Once sensitized, affected horses cough and have nasal discharge associated with progressive airway obstruction from a neutrophilic exudate, mucus hyperproduction, airway hyperreactivity and bronchospasm. Recurrent episodes of inflammation lead to smooth muscle hyperplasia, fibrosis and irreversible airway remodeling [3–6]. Severe equine asthma is responsive to environmental improvement and anti-inflammatory and bronchodilatory therapy, but is considered to be irreversible once airway remodeling has started [3]. Nevertheless, affected animals may have periods of clinical remission followed by periods of exacerbation over months to years.

Development of severe equine asthma likely involves genetic predisposition in addition to environmental triggers [7]. Findings have suggested that inheritance patterns are complex, implying genetic heterogeneity [8]. A significant association between susceptibility and paternal haplotype was proposed [9, 10], and specific regions on equine chromosomes 13 and 15 containing genes such as the *interleukin* (*IL*)*4* and *IL21* receptors were associated with increased risk of developing severe asthma in some equine kindreds [10, 11]. Copy number loss of a region on chromosome 5 including the gene *NME7* involved in ciliary function was more frequent in asthmatic than non-asthmatic horses [12]. However, strong evidence for a role of specific gene variations remains sparse.

The airway epithelium is the first barrier to inhaled substances, and includes multiple cell types such as ciliated and non-ciliated (club or Clara) cells, mucus producing goblet cells and precursor cells. It is thought that in severe equine asthma airway epithelial cells bind inhaled microbial components via pattern recognition receptors (PRR), which initiates an innate immune response with synthesis of inflammatory cytokines and chemokines [13]. In turn, inflammation of the epithelium results in generation of reactive oxygen metabolites, exosomes and proteases that injure epithelial cells and induce proliferation of airway smooth muscle cells, goblet cell hyperplasia, epithelial cell metaplasia and cell death [5, 14, 15]. In order for the epithelium to resume specialized barrier functions, cells need to regenerate with precise migration, proliferation and differentiation. Club cells, in particular, are markedly reduced in equine asthmatic airways resulting less anti-inflammatory secretoglobin 1A1 (SCGB1A1) in airway secretions [16, 17]. It is postulated here that repeated epithelial cell inflammation and injury results in progressively impaired regeneration of a fully functional epithelial barrier.

There are many proposed classification schemes for human asthma. According to most schemes, severe equine asthma is most similar to severe human adult or late onset asthma, which is distinct from childhood, allergic, exercise-induced and some other forms of human asthma [1, 18–20]. Phenotypes of human adult asthma are categorized according to age at onset, clinical characteristics, type of airway inflammation and response to therapy [19]. Severe human adult asthma is associated with airflow obstruction and most often neutrophilic inflammation, although eosinophilic and pauci-granulocytic inflammation is also observed [21]. Neither severe human adult asthma nor severe equine asthma is typically dominated by a Th2 immune response [3, 20]. It is difficult to investigate the pathogenesis of asthma in humans, and many inferences are based on nasal or sputum rather than bronchial or bronchiolar samples since the latter are difficult to obtain. Mice systemically sensitized to foreign antigen and then challenged by inhalation are widely used as models of human asthma, but recapitulate neither remission/exacerbation nor neutrophilic inflammation [22].

We hypothesized that the bronchial epithelial response to an inhaled challenge is different in asthmatic and non-asthmatic individuals. To address this hypothesis we designed a paired pre- and post-challenge study that accounts for individual variability in genetically heterogeneous animals, and obtained bronchial biopsy samples from affected and control animals that were processed for RNA sequencing and results analyzed.

## Methods

### Animals and procedures

**Fig. 1:**
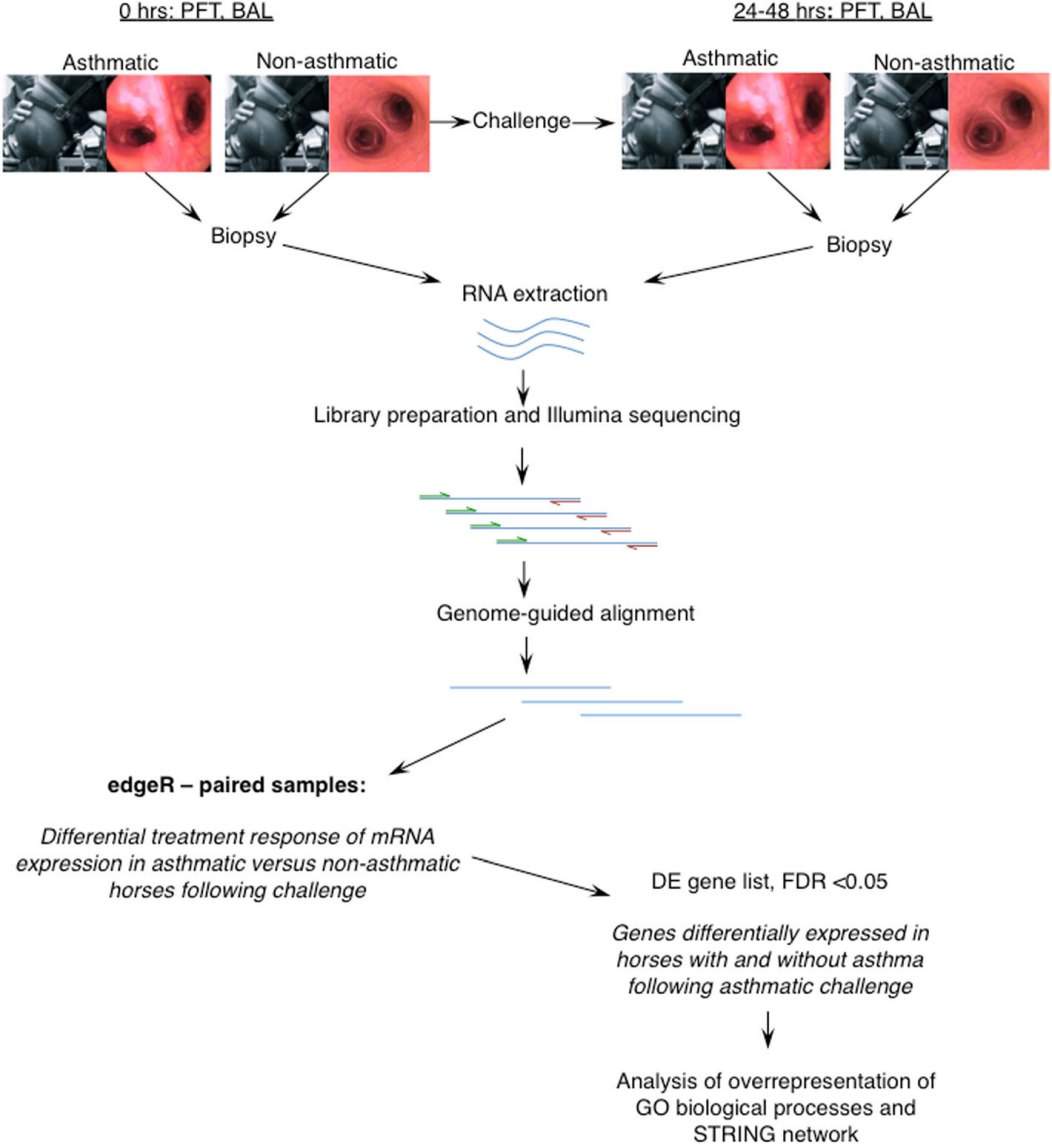
Outline of study design and analysis

Details of study design and analysis are presented in Fig. 1. Six horses with and seven horses without asthma had similar mean ages of 15 and 12 years (*p* = 0.352, unpaired t test), respectively, and each group included a variety of breeds. All were maintained for >6 months outdoors prior to study. Horses with historical asthma had been affected for 2 to 6 years, and were free of clinical respiratory disease during at least 6 months prior to study. All animals were placed in a dust-free indoor environment for 24 h, and thereafter physical examination, pulmonary function test (PFT) and bronchoalveolar lavage (BAL) were performed. During physical examination, respiratory rate, nasal discharge, presence and severity of expiratory lift, nasal flaring, tracheal sounds, bronchial tones, crackles, wheezes, cough and chest resonance were assessed according to a preset scale yielding a clinical score between 0 and 26. Pulmonary function data were derived from integration and analysis of airflow data and corresponding transpulmonary pressure. For PFT, non-sedated horses were restrained in stocks and fitted with a mask attached to a heated pneumotachograph. Airflow data were captured and fed through a transducer to integrate the flow signal and derive volume measurements. An esophageal balloon catheter was placed midthorax and attached to a transducer at the proximal end to estimate pleural pressure. Volume and pressure data were analyzed via respiratory loop analysis to derive values for pleural pressure (PpI), dynamic compliance (Cdyn) and lung resistance (RL). During bronchoscopy, the appearance of the upper airways, trachea and main bronchi were visualized, and scored for presence and degree of erythema, edema, secretions, hemorrhagic exudate, and cough reflex. An endoscopic score between 0 and 15 was derived from these parameteres. Then, the bronchoscope was gently lodged in a 3rd to 5th generation bronchus, and two sequential aliquots of 200 mL of warmed saline were infused and re-aspirated. An aliquot of BAL fluid was analyzed by total nucleated cell counting and 200-cell differential counting of stained cytocentrifuge preparations. Between two and eight endoscopic biopsies were obtained for RNA-seq and histopathology. Horses were then exposed to dusty hay until respiratory impairment was apparent in asthmatic horses (range 1 to 3 days, average 2.2 days). Non-asthmatic horses were exposed to dusty hay for 3 days. At this time clinical examination, respiratory function testing and BAL were repeated. BAL and endoscopic biopsies were obtained from a contralateral lung lobe. At exacerbation, mean clinical and bronchoscopic scores in asthmatic horses had increased from a mean of 2.7 to 13.3, and from 2.2 to 9.7, respectively. Non-asthmatic horses had mean clinical and bronchoscopic scores of 1.6 and 2.6 prior to challenge, and 0.4 and 1.9 post-challenge, respectively (Fig. 3 and Additional file 1: Table S1). The average change in PpI was 7.92 cm H_2_O in asthmatic horses, and −0.82 cm H_2_O in non-asthmatic horses. All procedures were approved by the Institutional Animal Care Committee of the University of Guelph (protocol R10–031) and conducted in compliance with Canadian Council on Animal Care guidelines. Changes in pulmonary function and BAL fluid composition between the two groups following an asthmatic challenge were analyzed by taking the differences between “after” and “before” values for each horse and testing with Welch’s t test for significant association with presence of asthma.

Changes in pulmonary function and BAL fluid (BALF) composition between the two groups following an asthmatic challenge were analyzed by taking the differences between “after” and “before” values for each horse and testing with Welch’s t test for significant association with presence of asthma. Graphs and *p*-values were generated using Prism 6.0a (GraphPad, La Jolla, CA) and unpaired t-tests with correction for multiple comparisons by the Holm-Sidak method with alpha = 5.000%. Significance threshold was set at *p* < 0.05. Complete information on horses and clinical tests is in Additional file 1: Table S1.

### RNA extraction, library preparation and sequencing

Total RNA was extracted from endobronchial biopsies (Qiagen, Toronto, ON). Quality and concentration of RNA were determined with the Bioanalyzer RNA Nanochip (Agilent, ON) and gel electrophoresis. Only samples with RNA integrity number > 6.9 and little to no degradation apparent on electrophoretograms were accepted. RNA-seq unstranded library preparation and sequencing were performed at The Centre for Applied Genomics (TCAG; Toronto, ON) using the Illumina TruSeq RNA sample preparation and sequencing protocol following the manufacturer’s guidelines (Illumina, San Diego, CA). Briefly, for each sample, approximately 1 μg of non-degraded, high quality total RNA was enriched for poly-A RNA, fragmented into 200 to 300 bases, and converted to double stranded cDNA libraries. Illumina adapters were ligated to the ds-cDNA and PCR-amplified for 14 cycles. Barcoded primers were then added to each sample to allow sequencing in the same lane and detection of individual samples in the sequence data. Final RNA libraries were quantified (KAPA Library Quantification kit, Kapa Biosystems, Wilmington, MA) prior to pooling and sequencing. Illumina flow cell was prepared and sequenced on an Illumina HiSeq 2500 instrument in 5 lanes following the manufacturer’s instructions to generate paired-end reads of 100-bases.

### Genome-guided RNA alignment

Raw read quality was assessed using FastQC software version 0.10.1 [23] and aligned to the horse reference genome [24] (Ensembl v70) with STAR version 2.4 [25]. The STAR_pass2 alignment protocol was followed including these adaptations: horse Ensembl version 70 GTF annotation file for first- and second-pass, and the junction SJ.tab file generated by STAR for the second-pass after non-canonical junctions were removed. Default settings were used except for: --runThreadN 8 --outFilterScoreMinOverLread 0.5 --outFilterMatchNminOverLread 0.5. Read counts were generated from STAR alignment files using HTSeq version 0.6.1p1 [26] with settings -s no -f bam -r name.

### Differential gene expression

Differential expression (DE) analysis was performed in R, version 3.2.1 [27], with the edgeR package version 3.10.2 [28–30]. A paired DE analysis was performed to assess changes between groups (asthmatics versus non-asthmatics) and within groups (before versus after challenge). EdgeR analysis was based on section 3.5 of the edgeR user’s guide (last revised April 10, 2017). Briefly, the minimum count number was set at 1 read per million in at least 3 samples. Normalization factors and effective library size were applied, duplicates were removed and dispersion was estimated using the “estimateGLMCommonDisp”, “estimateGLMTrendedDisp” and “estimateGLMTagwiseDisp” functions. The model matrix was designed as: ~group + group:horse + group:challenge, where “group” refers to non-asthmatic and asthmatic groups, “horse” refers to each individual horse, and “challenge” refers to samples before and after the asthmatic challenge. Fit of the generalized linear model and tests for differences in expression were performed with the “glmFit” and “glmLRT” functions, respectively and the following contrast was used to compare asthmatic and non-asthmatic horses: glmLRT(fit, contrast = c(0,0,0,0,0,0,0,0,0,0,0,0,0,-1,1)). GC content bias was assessed using EDAseq [31], but need for normalization was not indicated. Statistical significance was set at a false discovery rate (FDR) <0.05.

### Immunohistochemistry

The protein product of four genes with significant up- or down-regulation was assessed by immunohistochemistry (IHC). Confirmation of protein expression for a group of genes was deemed sufficient as a proxy to confirm the correctness of sequencing, alignment and statistical workflow. The genes were selected based on significant differential expression between asthmatic and non-asthmatic horses, availability of cross-reactive antibodies and potential roles in asthma pathogenesis. Antibodies were initially tested in Western blots with equine tissue samples to verify that a single protein product of appropriate size was detected (data not shown). Tumor necrosis factor receptor superfamily member 12A (TNFRSF12A or TWEAKR, tumor necrosis factor-like weak inducer of apoptosis receptor), patched-1 (PTCH1), cell division cycle 25 homolog A (CDC25A) and interleukin 8 (IL8) proteins were assessed in biopsies fixed in formalin and routinely sectioned and processed. Antibody reactivity was first assessed by western blot (WB) analysis against horse serum or lung protein extracts (Additional file 2: Figure S1). Proteins were separated in 12% (*w*/*v*) SDS-polyacrylamide gels (TGX Stain-Free FastCast premixed acrylamide solutions; Bio-Rad, Mississauga, ON) under reducing conditions. Proteins were then electro-transferred to PVDF membranes using the Trans-Blot Turbo Transfer System (Bio-Rad). Membranes were blocked in 5% BSA solution before immunoblotting with polyclonal rabbit anti-human TWEAKR (Biorbyt, Berkeley, CA), PTCH1 (C-terminal region; Aviva Systems Biology, San Diego, CA) and CDC25A (Abcam, Toronto, ON), and polyclonal rabbit anti-horse IL8 (MyBioSource, Inc., San Diego, CA). Membranes were then incubated with horseradish peroxidase (HRP)-conjugated goat anti-rabbit secondary antibody (DAKO, Mississauga, ON) and exposed with Clarity Western ECL Substrates (Bio-Rad). Images were captured with a ChemiDoc imaging system (Bio-Rad). If bands of expected size were present, antibodies were applied in immunohistochemistry (IHC) to 3–5 μm thick sections placed on charged glass slides, de-paraffinized in xylene, rehydrated in alcohol, incubated with dual endogenous enzyme blocker and serum-free protein blocker (both DAKO). Slides were then sequentially incubated with the above primary antibodies, Envision Dual Link System-HRP (DAKO) and Nova Red chromogen (Vector Laboratories, Burlingame, CA), and counterstained with hematoxylin. Negative control sections for each IHC analysis were prepared in the same manner except the primary antibody was omitted.

### Protein network and gene ontology analysis

Gene products were searched for known and predicted protein interactions in Cytoscape version 3.4.0 [32] using the Search Tool for the Retrieval of Interacting Genes/Proteins (STRING) database [33] and *string-db* plugin [34] within Cytoscape. Horse Ensembl ID were converted to human ID using Biomart [35, 36] and to gene symbols directly in Cytoscape through the STRING database (Additional file 1: Table S2). When multiple horse Ensembl IDs had identical human symbols, redundant symbols were removed. The confidence score cut-off applied for interactions was 0.4 (medium confidence). Single nodes, doublets and triplets detached from the main network cluster were removed, and network analysis was performed. Node color and size was determined based on betweenness centrality (BC) and degree, respectively. Confidence of interactions was displayed with different intensity of edge color.

Gene ontology (GO) overrepresentation analysis of biological function was performed with *Protein ANalysis THrough Evolutionary Relationships* (PANTHER) software version 10.0 [37] with significance threshold set at *p* < 0.05 (including Bonferroni adjustment). The analysis was performed using annotations for *Homo sapiens* by converting the equine gene symbols into human gene symbols prior to analysis. Species to be analyzed was then identified as human, and information on protein function was assigned to candidates according to prediction in NCBI or UniProt databases.

## Results

### Induction of asthma

**Fig. 2:**
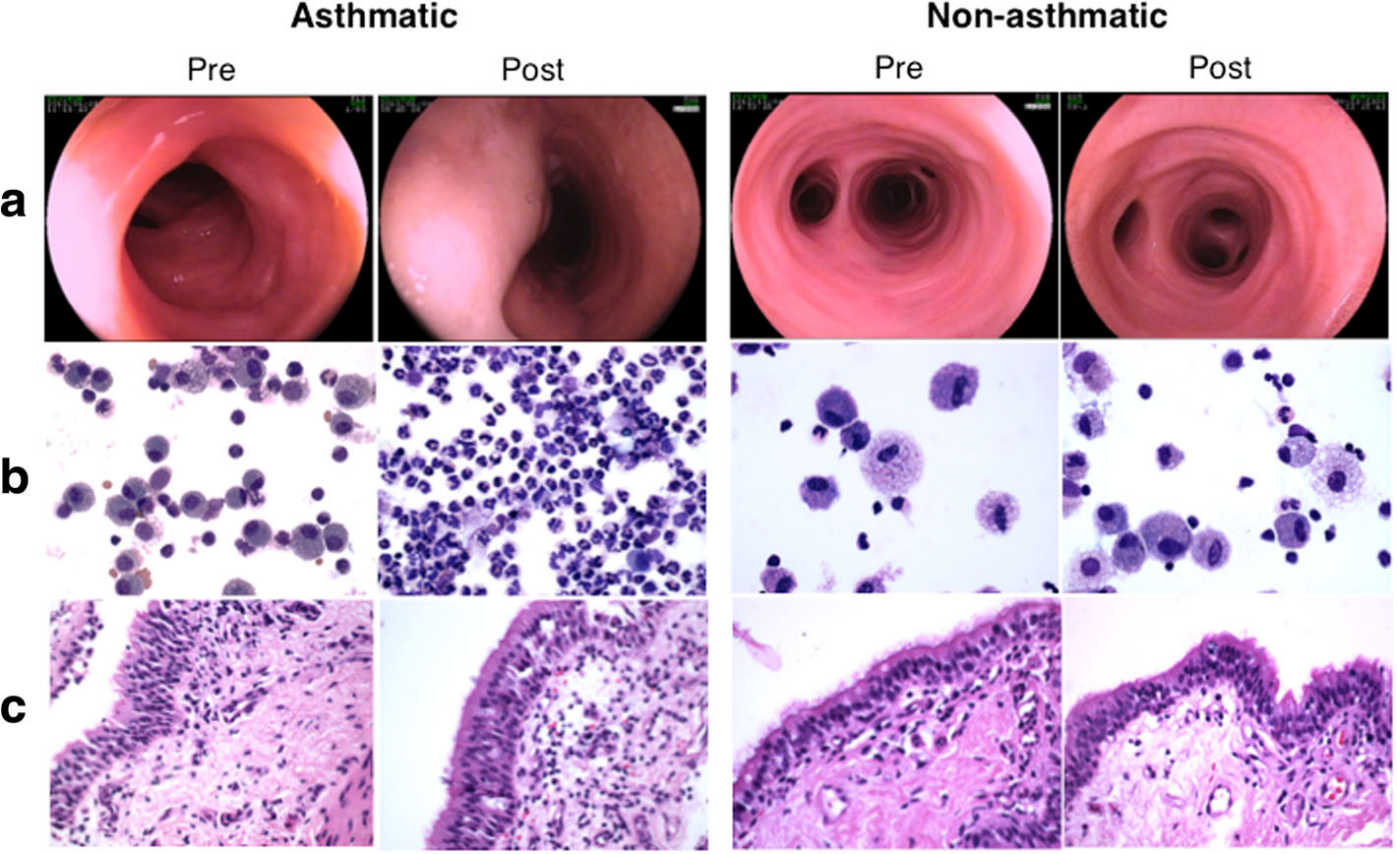
Endoscopic appearance of bronchi (**a**), BAL lavage cell yield (**b**) and histological appearance of bronchial biopsies (**c**). Asthmatic horses had bronchoconstriction and increased secretions in airways after asthmatic challenge, while changes in horses without asthma consisted of mild bronchoconstriction (**a**). In asthmatic horses, cell concentration and proportion of neutrophils was increased in BAL fluid after challenge (**b**), and in bronchial biopsies epithelial basophilia and influx of submucosal leukocytes was evident (**c**)

**Fig. 3:**
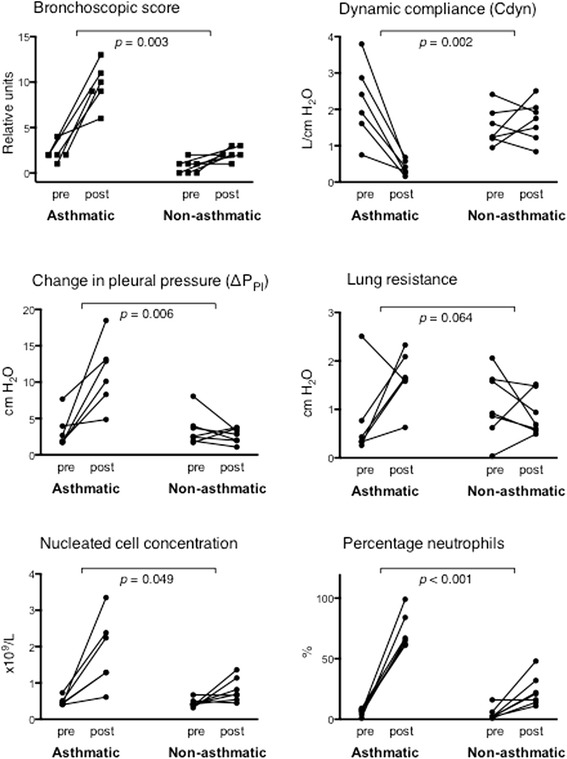
The individual change from pre- to post-challenge bronchoscopic score, pleural pressure, alveolar leukocyte concentration, proportion of neutrophils, and dynamic compliance differed significantly between asthmatic and control horses, while the change in lung resistance did not have a uniform pattern

Following exposure to inhaled challenge material, severe bronchoconstriction and profound airway secretions were apparent in asthmatic but not non-asthmatic horses (Fig. 2a), and cell concentration and the proportion of neutrophils were increased in bronchoalveolar lavage (BAL) fluid (Fig. 2b). Bronchial biopsies showed submucosal edema and an influx of leukocytes (Fig. 2c). Changes in BAL fluid cell and tissue composition in non-asthmatics were mild or absent. As a group, asthmatic horses had significantly higher bronchoscopic scores, pleural pressure, BAL nucleated cell concentration and percent neutrophils, and significantly lower dynamic compliance after asthmatic challenge than non-asthmatics (Fig. 3). Complete data are in Additional file 1: Table S1.

### Differential expression analysis

**Fig. 4:**
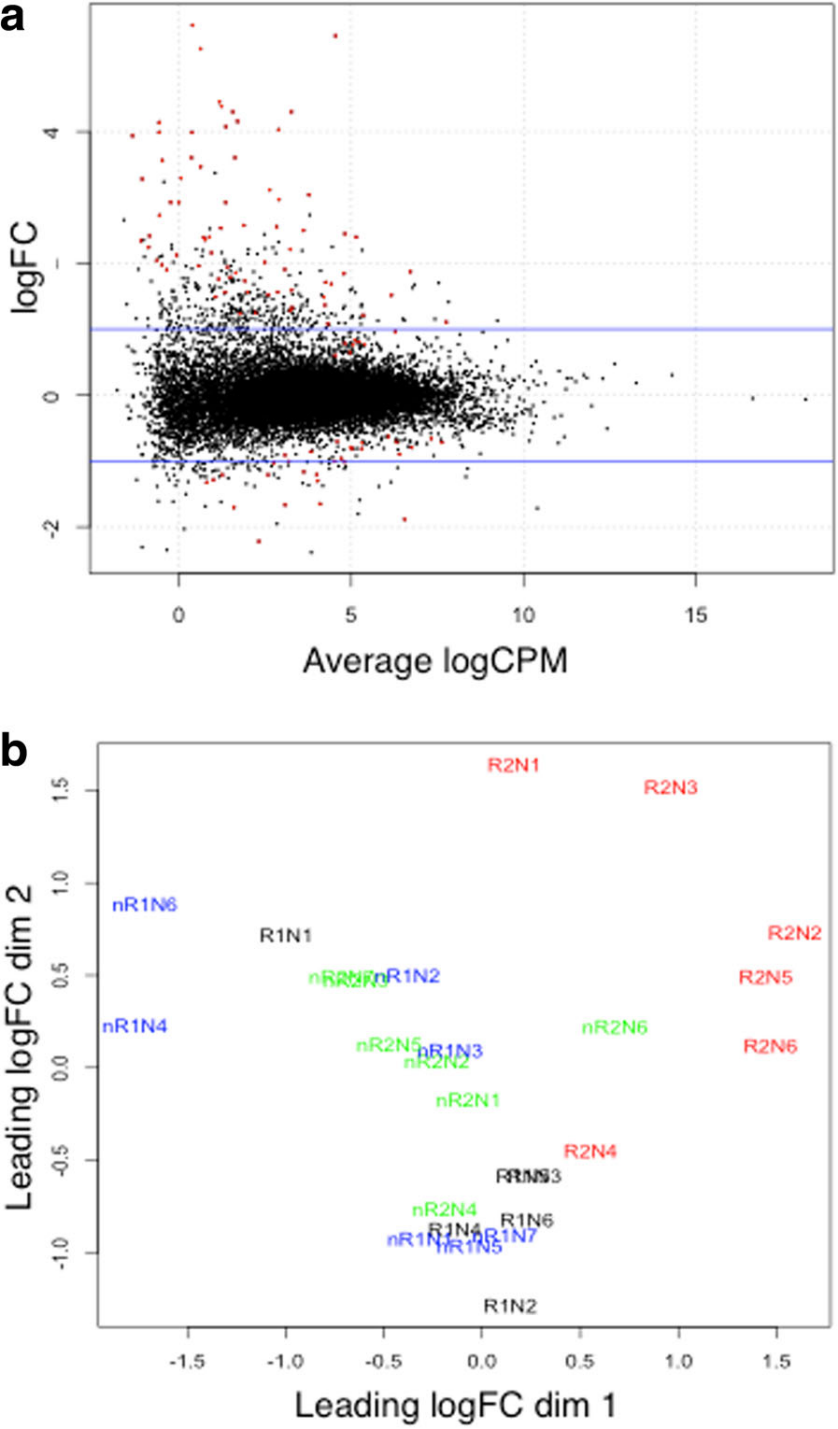
EdgeR smear plot showing the log2 fold-change (FC, y-axis) versus the average log2 count per million (CPM, x-axis) of the change in gene expression due to challenge in all horses (**a**). Horizontal blue lines delineate 1-fold change and each point represents one gene. Differentially expressed genes are indicated in red (FDR <0.05). Genes with positive log2FC were up-regulated in asthmatic compared to non-asthmatic horses, while genes with negative FC were down-regulated. The majority of genes expressed differentially between the two groups are up-regulated. Leading logFC plot (**b**) shows individual horses with asthma before (black) and after (red) challenge, and horses without asthma before (blue) and after (green) challenge. No clustering was observed for non-asthmatic horses, but post- challenge samples from asthmatic horses are located distant from other samples

The results of mapped RNA-seq reads for individual horses are summarized in Table 1. Analysis identified 111 genes differentially expressed (DE) between horses with and without asthma as a result of challenge (Fig. 4a). Significantly differentially expressed genes pertain to the epithelium and include keratin-related genes (identified as potential *Keratin [KRT] 6* based on human homologues ENSG00000185479, and *KRT17*), genes linked to matrix metalloproteinases (*MMPs*), inflammation (*Toll-like receptor 4* (*TLR4*), and others), neutrophil chemotaxis (*IL8, C-X-C chemokine receptor type 2* (*CXCR2*)), coagulation and hemostasis (such as *Pleckstrin* (*PLEK*)), cell proliferation (*CDC25A*), apoptosis (such as *BCL2 interacting killer* [*BIK*]) and others. Six of 30 down-regulated genes function in regulation of the circadian rhythm. Identity and details of DE genes are in Additional file 1: Table S2.

**Table 1:** Summary of RNA-seq reads mapped to the horse genome

Sample	Total reads	Total mapped	Uniquely mapped	Multiple mapped
R1N1	30,751,400	29,497,889 (95.93%)	28,289,828 (92%)	1,208,061 (3.93%)
R2N1	29,096,094	27,924,026 (95.97%)	26,796,723 (92.1%)	1,127,303 (3.87%)
R1N2	32,809,322	31,473,113 (95.92%)	30,216,857 (92.10%)	1,256,256 (3.82%)
R2N2	36,872,731	35,096,310 (95.18%)	33,642,523 (91.24%)	1,453,787 (3.94%)
R1N3	33,871,258	32,377,015 (95.59%)	31,022,612 (91.59%)	1,354,403 (4.0%)
R2N3	33,703,288	32,248,448 (95.68%)	30,927,028 (91.76%)	1,321,420 (3.92%)
R1N4	28,780,180	27,551,694 (95.73%)	26,196,530 (91.02%)	1,355,164 (4.71%)
R2N4	37,016,186	35,272,681 (95.29%)	33,603,034 (90.78%)	1,669,647 (4.51%)
R1N5	44,838,345	42,510,917 (94.81%)	40,489,583 (90.30%)	2,021,334 (4.51%)
R2N5	48,307,368	45,988,565 (95.2%)	43,877,495 (90.83%)	2,111,070 (4.37%)
R1N6	47,157,281	44,765,270 (94.93%)	42,647,415 (90.44%)	2,117,855 (4.49%)
R2N6	51,788,389	49,290,097 (95.18%)	47,078,696 (90.91%)	2,211,401 (4.27%)
nR1N1	36,071,224	34,556,532 (95.8%)	32,999,057 (91.48%)	1,557,475 (4.32%)
nR2N1	37,488,512	35,847,340 (95.63%)	34,102,178 (90.97%)	1,745,162(4.66%)
nR1N2	34,952,730	33,371,917 (95.48%)	31,645,390 (90.54%)	1,726,527 (4.94%)
nR2N2	38,173,366	36,621,999 (95.93%)	34,949,535 (91.55%)	1,672,464 (4.38%)
nR1N3	37,452,422	36,033,693 (96.21%)	34,446,130 (91.97%)	1,587,563 (4.24%)
nR2N3	39,708,395	38,014,973 (95.73%)	36,309,547 (91.44)	1,705,426 (4.29%)
nR1N4	35,781,991	34,339,176 (96.37%)	32,692,804 (91.37%)	1,646,372 (5.0%)
nR2N4	34,901,655	33,484,332 (96.34%)	31,867,656 (91.31%)	1,616,676 (5.03%)
nR1N5	31,277,591	30,122,423 (96.3%)	28,941,665 (92.53%)	1,180,758 (3.77%)
nR2N5	35,994,809	34,677,174 (96.34%)	33,341,679 (92.63%)	1,335,495 (3.71%)
nR1N6	29,463,977	28,177,878 (95.64%)	26,861,858 (91.17%)	1,316,020 (4.47%)
nR2N6	35,834,021	34,358,029 (95.87%)	32,871,846 (91.73%)	1,486,183 (4.14%)
nR1N7	34,515,301	33,067,972 (95.81%)	31,655,814 (91.72%)	1,412,158 (4.09%)
nR2N7	25,962,392	24,914,311 (95.97%)	23,840,642 (91.83%)	1,073,669 (4.14%)

The relationship between paired samples from individual animals is shown in a multidimensional scaling (MDS) plot with the distance between pairs of samples corresponding to the average root mean square of the largest log2FC (leading logFC, Fig. 4b). Post-challenge samples from asthmatic animals were distinctly distant from those of pre-challenge asthmatic and non-asthmatic animals. This implies that within the asthmatic group differential expression of genes was greater than biological variance, and that most of the DE genes originated from asthmatic animals. The biological coefficient of variance (BCV) was calculated to determine how much the variance in counts exceeded that which would arise from Poisson counts alone [29]. The BCV for RNA-seq analysis of genetically identical organisms is typically around 0.1 [29] while in this study the BCV was 0.23 (data not shown). This high value reflects the biological variance as expected from outbred individuals, and also emphasizes the benefit of a paired sample design to correct for inter-individual variation.

**Fig. 5:**
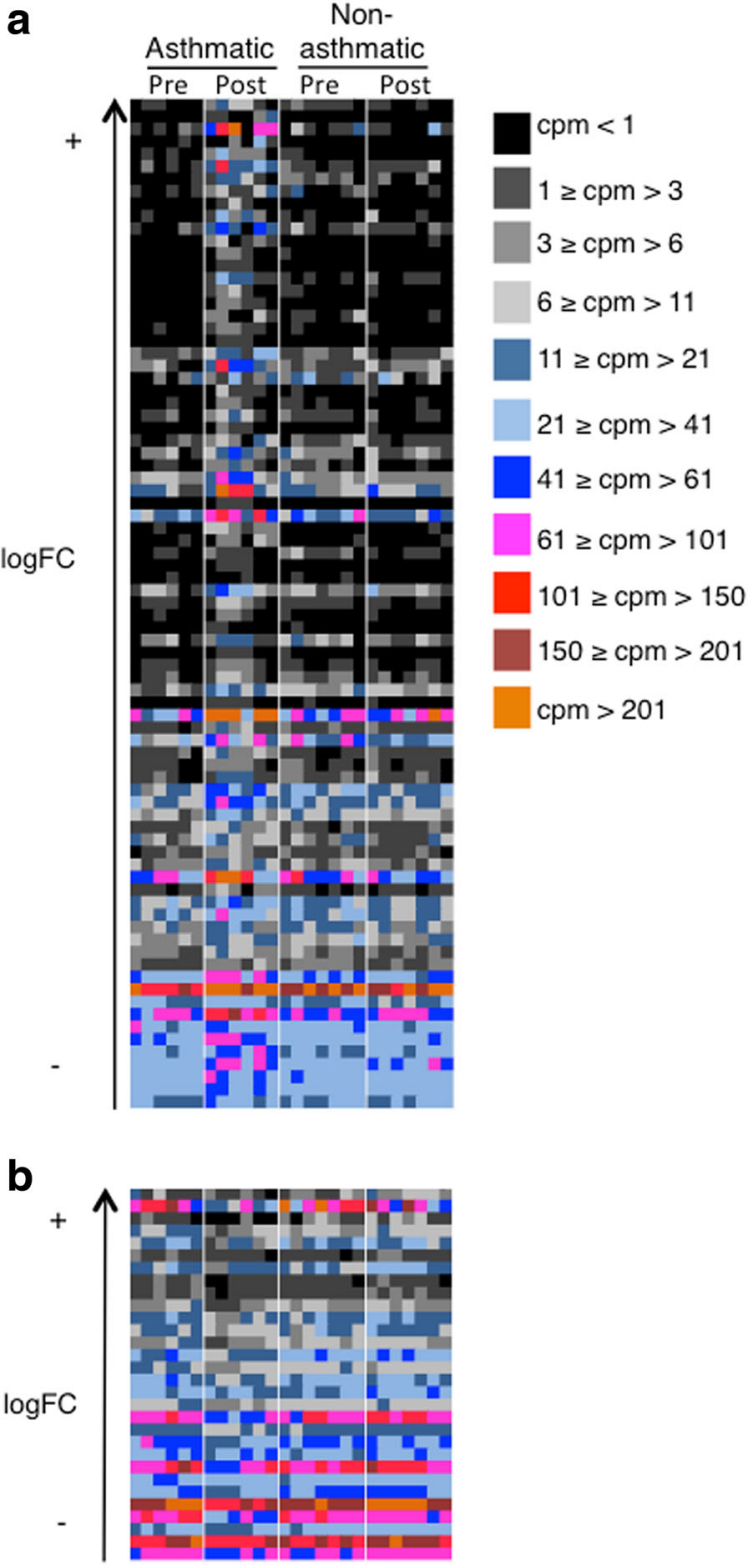
A heat map of differentially expressed genes significantly up- (**a**) and down-regulated (**b**) between asthmatic and non-asthmatic horses after challenge shows consistent change across individuals. Genes with positive log2FC were up-regulated in asthmatic compared to non-asthmatic horses, while genes with negative FC were down-regulated. Level of expression is expressed as cpm and ordered from highest (top) to lowest log2 fold-change (logFC, bottom). Significance threshold was set at FDR <0.05

**Fig. 6:**
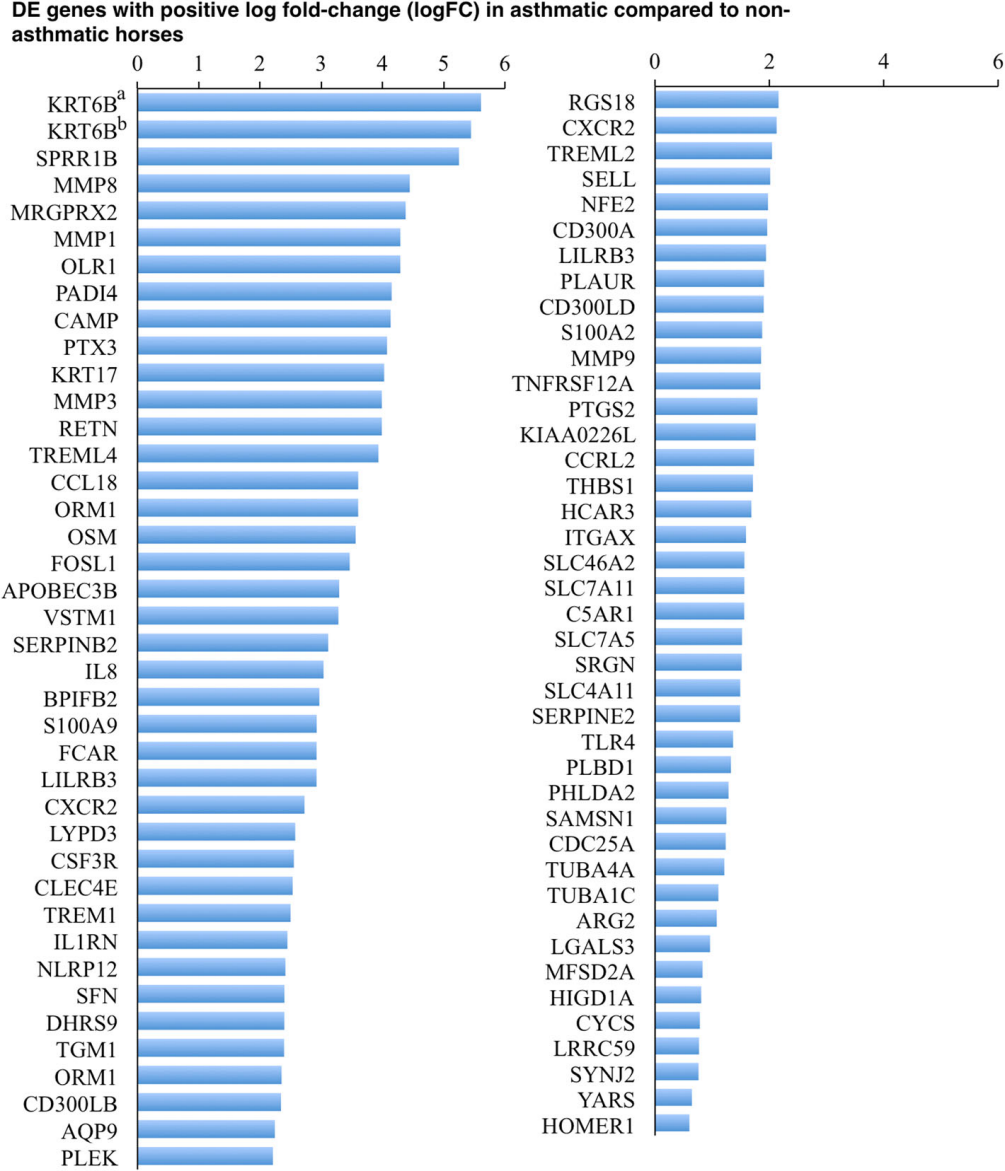
Stacked bar chart of positive log2 fold-change (logFC) for genes differentially expressed between asthmatic and non-asthmatic horses in response to challenge. ^a^ ENSECAG00000014899; ^b^ ENSECAG00000017229

**Fig. 7:**
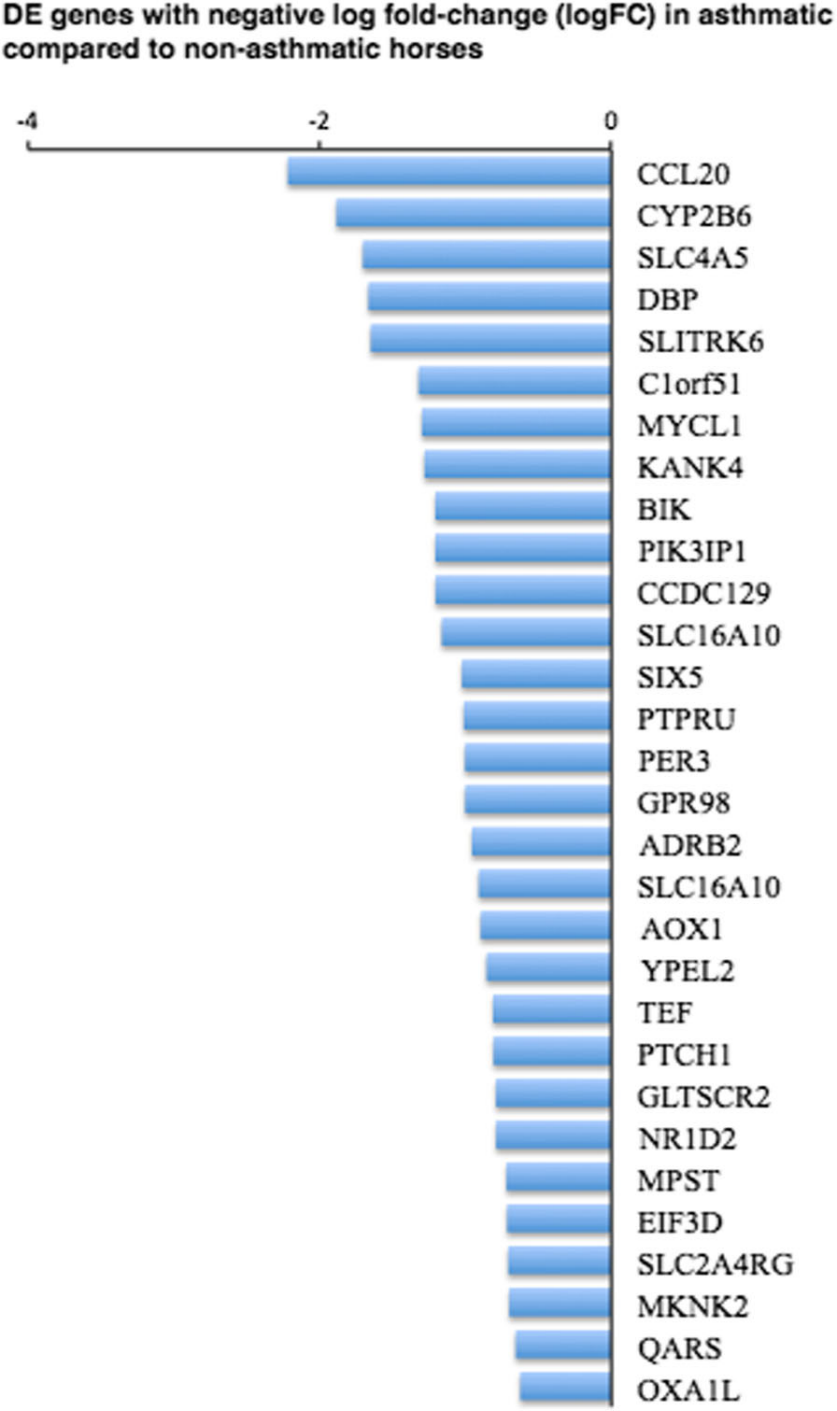
Stacked bar chart of negative log2 fold-change (logFC) for genes differentially expressed between asthmatic and non-asthmatic horses in response to challenge

A heat map of counts per million (CPM) of DE genes for each sample (Fig. 5) shows that there is a wide distribution of change in expression (logFC), an inverse relationship of CPM with log FC, and consistency of change across individuals. The magnitude of the log fold change of significantly up-regulated genes in asthmatic horses after challenge ranged from 0.6 to 5.6 (Fig. 6), and that of significantly down-regulated genes from −0.62 to −2.2 (Fig. 7).

### Protein expression

**Fig. 8:**
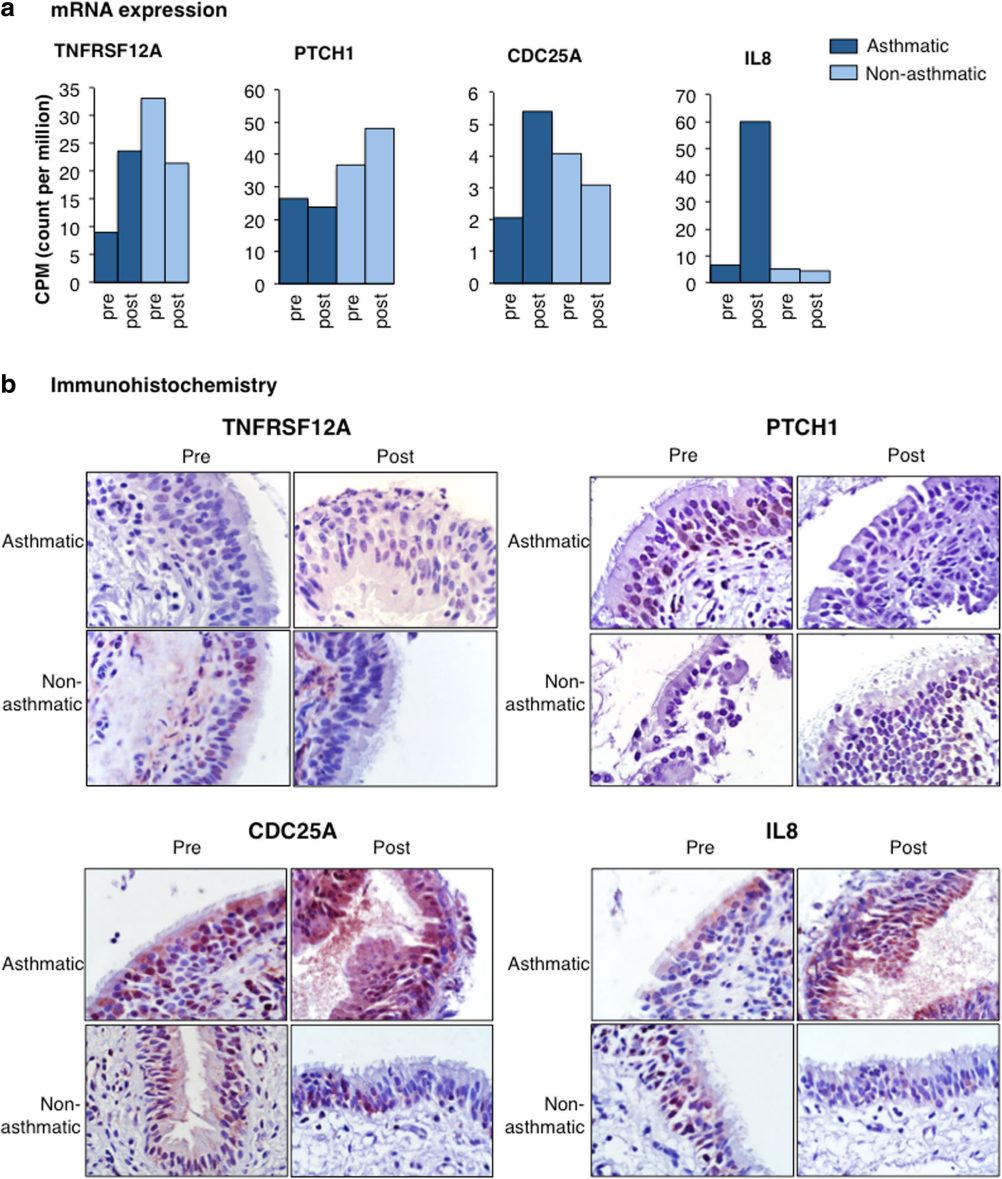
Gene (**a**) and protein expression (**b**) of TNFRSF12A, PTCH1, CDC25A and IL8 in bronchial biopsies from horses with and without asthma. TNFRSF12A, CDC25A and IL8 mRNA, expressed as counts per million (CPM), was up-regulated in asthmatic and down-regulated in non-asthmatics following challenge, while PTCH1 was slightly down-regulated in asthmatics and up-regulated in non-asthmatics. (**b**) IHC results approximated gene expression with a relative increase in TNFRSF12A, CDC25A and IL8 immunoreactivity and decrease in PTCH1 immunoreactivity in asthmatic animals

Specific RNA-seq results were further investigated in biopsy tissues by IHC. Expression of *TNFRSF12A, CDC25A* and *IL8* mRNA was markedly increased in asthmatic and decreased in non-asthmatic horses after challenge, while *PTCH1* mRNA was slightly decreased (Fig. 8a). Immunohistochemical results representative of each group showed more intense reactivity for TNFRSF12A, CDC25A and IL8 protein in tissues from asthmatic than non-asthmatic horses after challenge (Fig. 8b). TNFRSF12A staining was moderately intense throughout the epithelium of asthmatics after challenge and only present in individual epithelial and sub-epithelial cells from non-asthmatics. CDC25A reactivity was intense in epithelium of asthmatic animals, and less prominent in tissue from non-asthmatic animals, in particular after challenge. IHC results for IL8 also showed marked increase after challenge in asthmatic but not non-asthmatic animals. PTCH1 staining was less abundant in asthmatic than non-asthmatic animals after challenge.

### Gene ontology analysis

PANTHER analysis of GO overrepresentation for biological processes (GOBP) using the *Homo sapiens* database identified significantly overrepresented gene sets among up- and down-regulated genes, listed in Table 2 with associated gene names. The most specifically involved gene sets concerned neutrophil chemotaxis (GO:0030593), immune response (GO:0006955), inflammatory response (GO:0006954), secretion (GO:0046903), positive regulation of blood coagulation (GO:0030194), positive regulation of apoptotic signaling pathway (GO:2,001,235), positive regulation of response to external stimulus (GO:0032103) and regulation of immune system process (GO:0002682) for up-regulated genes, and rhythmic process (GO:0048511) for down-regulated genes.

**Table 2:**
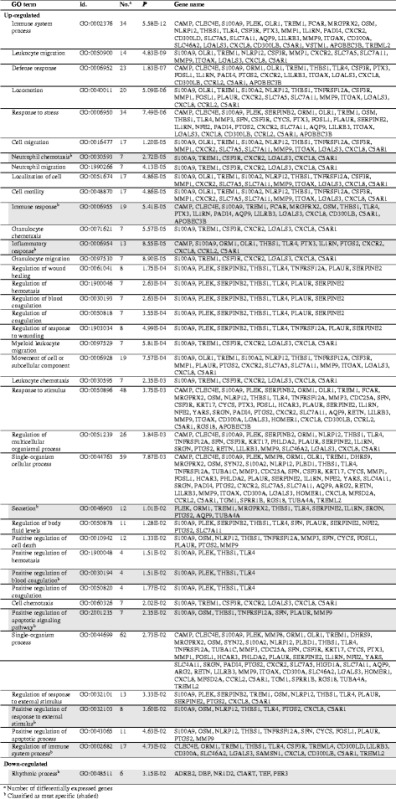
Significantly over-represented biological processes identified by GO analysis

^a^ Number of differentially expressed genes

^b^ Classified as most specific (shaded)

Up-regulated genes shared between the most specific gene-sets are shown in Table 3 along with evidence for their association with asthma and their known functions. *S100 Calcium Binding Protein A9 (S100A9)* was the only gene that contributed to all gene sets, and has been associated with asthma in mice [38]. All genes associated with 4 or more gene sets have also been associated with asthma in humans or mice and include *S100A9, thrombospondin 1 (THBS1), TLR4, IL8, complement component 5a receptor 1 (C5AR1), MMP9, NLR Family, Pyrin Domain Containing 12 (NLRP12)* and *triggering receptor expressed on myeloid cells 1 (TREM1)* [20, 38–48]. Other genes associated with 3 or fewer gene sets have also been associated with asthma such as *Plasminogen Activator, Urokinase Receptor* (*PLAUR*) and *Serpin Family E Member 2* (*SERPINE2*), and several additional genes were first identified here.

**Table 3:** The function of genes with overrepresented GO terms

Gene symbol	Positive regulation of immune system process	Immune response	Inflammatory response	Positive regulation of response to external stimulus	Secretion	Positive regulation of apoptotic signalling pathway	Neutrophil chemotaxis	Positive regulation of blood coagulation	Association with asthma	Protein function (NCBI or Uniprot; *Homo sapiens*)
S100A9	✓	✓	✓	✓	✓	✓	✓	✓	Promotes inflammation with and without TLR4 interactions in a mouse model [38]	Regulation of cellular processes such as cell cycle and differentiation. Antifungal and antibacterial activity
THBS1	✓	✓	✓	✓	✓	✓	×	✓	Central in a network linked to pulmonary response to oxidative stress in asthma [39]	Cell-to-cell and cell-to-matrix interactions
TLR4	✓	✓	✓	✓	✓	×	×	✓	Linked to allergic asthma in a mouse model [41]	Pathogen recognition and innate immunity
CXCL8	✓	✓	✓	✓	×	×	✓	×	Biomarker of neutrophilic asthma in adult [20]	Chemoattractant and angiogenic factor
C5AR1	✓	✓	✓	✓	×	×	✓	×	Linked to regulation of airway remodelling [42]	Receptor for C5a, ligand with chemotactic and inflammatory activity
MMP9	✓	✓	✓	×	✓	✓	×	×	Increased in severe asthma, involved in Th2 response and EMT in human bronchial epithelial cells [43, 44]	Matrix metalloproteinase, breakdown of extracellular matrix
NLRP12	✓	✓	✓	✓	✓	×	×	×	Increased expression in asthmatics on high-fat diet. No observed effect in allergic airway disease models [45–47]	Anti-inflammatory molecule
TREM1	✓	✓	×	×	✓	×	✓	×	Correlates with clinical stage and neutrophils in human asthmatics [48]	Amplifies neutrophil and monocyte inflammatory response
PLEK	✓	×	×	×	✓	×	×	✓	NA^a^	EMT in bronchial epithelial cells [47]
LGALS3	✓	✓	×	×	×	×	✓	×	Key role in inflammation and airway remodelling in murine models of allergic asthma [95–97]. Decreased in sputum of neutrophilic asthmatics compared to mixed neutrophilic and eosinophilic asthmatics [98]	Involved in apoptosis, innate immunity, cell adhesion and T-cell regulation Antimicrobial activity
OSM	×	✓	×	✓	×	✓	×	×	Part of IL6 family of cytokines, OSM specifically is increased in asthmatics and correlates with irreversible airway obstruction [99]. OSM was associated with asthma but not atopy in children [100]	Inhibition of proliferation, regulation of production of other cytokines
PTGS2	×	×	✓	✓	✓	×	×	×	Polymorphism in the gene associated with asthma and atopy in children [101]	Prostaglandin biosynthesis
ORM1	✓	×	✓	×	✓	×	×	×	NA	Acute-phase protein, potential immunosuppressive activity
IL1RN	×	✓	✓	×	✓	×	×	×	Sputum IL1RN to IL-1β ratio decreased in neutrophilic asthmatics compared to mixed neutrophilic and eosinophilic asthmatics [98]	EMT in bronchial epithelial cells [47] Modulates IL1-mediated inflammation and inhibits activity of IL1A & B
CSF3R	✓	×	×	×	×	×	✓	×	NA	Controls granulocyte production and activity.
CXCR2	×	×	✓	×	×	×	✓	×	Expression increased in sputum of patient with non-eosinophilic asthma [21]. Controversial role in asthma. CXCR2 antagonists alone do not improve clinical signs of asthma but reduce neutrophil number [21]. Combination of CXCR1 and CXCR2 antagonists promising for therapy [102]	IL8 receptor, neutrophil migration
CAMP	×	✓	✓	×	×	×	×	×	NA	Response to pathogens, Antibacterial, regulation of cell chemotaxis and inflammation.
PTX3	×	✓	✓	×	×	×	×	×	Expression increased in bronchial tissues of asthmatics and highly expressed in smooth muscle cells [103]	Up-regulated in response to inflammation in epithelial cells. Role in angiogenesis and tissue remodelling.
MRGPRX2	×	✓	×	×	✓	×	×	×	NA	IgE independent activation of mast-cells, leading to inflammation and smooth muscle cell contraction [104]. Mast cell-specific receptor, mediates allergic reactions.
AQP9	×	✓	×	×	✓	×	×	×	NA	Neutrophil regulation [105]; membrane channel
CLEC4E	✓	✓	×	×	×	×	×	×	NA	Inflammation
LILRB3	✓	✓	×	×	×	×	×	×	NA	Inhibits immune response
CD300LB	✓	✓	×	×	×	×	×	×	NA	Expressed by granulocytes [106]. Activates immune receptor
**Specific to one gene set**										
TNFRSF12A	×	×	×	×	×	✓	×	×	NA	May be involved in angiogenesis, proliferation and cellular adhesion
SFN	×	×	×	×	×	✓	×	×	NA	Regulation of protein kinase C [107] and epithelial cell growth through Akt/mTOR pathway when bound to KRT17 [108]
PLAUR	×	×	×	×	×	✓	×	×	Increased expression in vitro in bronchial epithelium from asthmatics [109]	Impaired wound repair process [109]. May be involved in plasminogen activation and ECM degradation
OLR1	×	×	✓	×	×	×	×	×	NA	Degradation of oxidized low-density lipoprotein
CCRL2	×	×	✓	×	×	×	×	×	Expressed in lung epithelial cells and up-regulated following LPS exposure. Potential role in initiation of allergic inflammation [110]	Unknown function, up-regulated in activated neutrophils
SERPINE2	×	×	×	×	✓	×	×	×	Urokinase inhibitor [111], overexpression in asthma and down-regulation correlated with improved FEV1 [112]	Serine protease inhibition
SRGN	×	×	×	×	✓	×	×	×	NA	Hematopoietic cell granule proteoglycan
TUBA4A	×	×	×	×	✓	×	×	×	NA	Component of microtubules
FCAR	×	✓	×	×	×	×	×	×	NA	Surface receptor of myeloid cells, regulation of immune defense processes
PADI4	×	✓	×	×	×	×	×	×	NA	Required for NET formation [79] in equine asthma [78], conversion of arginine to citrulline
APOBEC3B	×	✓	×	×	×	×	×	×	NA	Up-regulated by PKC and increased likelihood of mutations [113], RNA editing
TREML4	✓	×	×	×	×	×	×	×	NA	Immune response
CD300LD	✓	×	×	×	×	×	×	×	NA	Immune response
CD300A	✓	×	×	×	×	×	×	×	Co-aggregation of CD300A with CCR3 decreased clinical signs of asthma in a mouse model [114]	Regulation of mast cell response [115]. Surface protein on leukocytes involved in immune response
SLC46A2	✓	×	×	×	×	×	×	×	NA	Potential transporter function
SAMSN1	✓	×	×	×	×	×	×	×	NA	Cell proliferation, spreading and regulation of polarization
TREML2	✓	×	×	×	×	×	×	×	Associated with asthma but not atopy in children [100]	Surface receptor, role in innate and adaptive immune response

^a^ Not available

Among down-regulated genes, only the rhythmic process (GO:0048511) gene set was overrepresented. This gene set includes genes associated with asthma in humans and mice such as *adrenoceptor beta 2 (ADRB2), nuclear receptor subfamily 1 group D member 2 (NR1D2)* and *period circadian clock 3 (PER3)*, as well as genes that have not previously been linked to asthma such as *D site of albumin promoter (albumin D-box) binding protein (DBP)*, *circadian-associated repressor of transcription (CIART or CHRONO, ChIP-derived Repressor of Network Oscillator)* and *thyrotrophic embryonic factor (TEF)*.

### Protein network analysis

**Fig. 9:**
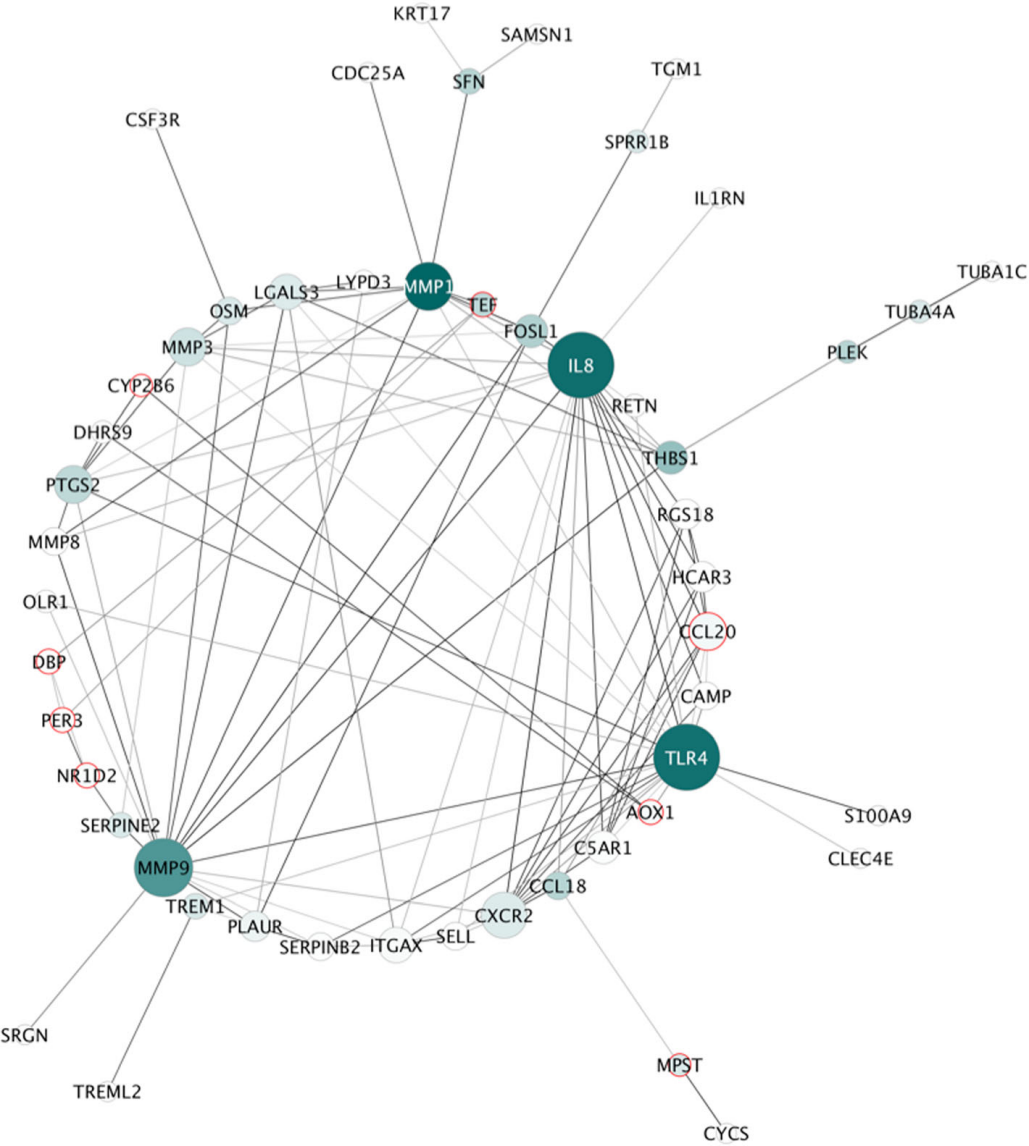
The main network cluster of genes DE between asthmatic and non-asthmatic horses after challenge. The network cluster is derived from 111 genes and contains 51 nodes each representing a protein, and 113 edges each representing an interaction between two proteins. Node color from white to green represents lowest to highest betweenness centrality (BC). The size of each node corresponds to the degree (number of connections). The color of edges represents the confidence of the interaction ranging from 0.4 (medium confidence, light gray) to 1 (highest confidence, black). Nodes with red borders have negative fold-change

Protein products of genes DE between groups in response to challenge identified multiple interactions with medium to high confidence (scores ranging from 0.4 to 1). The main protein interaction cluster derived from the 111 DE genes contained 51 nodes, each representing one protein and connected by 113 edges (Fig. 9). MMP1, IL8 and TLR4, followed by MMP9, had highest scores for betweenness centrality (BC), indicating they are most important for connections with other proteins. IL8, TLR4 and MMP9 had the highest number of direct connections (degree). S100A9, associated with all overrepresented gene sets (Table 3), is connected to the network through its predicted interaction with TLR4. In addition, MMP1 and THBS1 each connect two genes (Table 3) with potential role in severe equine asthma though not yet associated with asthma in humans or mice.

## Discussion

The goal of this study was to identify bronchial epithelial genes and pathways associated with severe asthma in horses. Although predisposition for development of severe asthma in horses is thought to be hereditary, inheritance is incompletely defined and thought to be complex [7]. Analysis of the bronchial epithelium aimed to capture the in situ genetic changes that characterize the pathogenesis of severe equine asthma, an approach difficult to apply in other species. Although components of the lung such as bronchioles, alveoli, interstitium and leukocytes are also affected in asthma, they were not specifically evaluated in this study. Biopsies consisted predominantly of epithelium, which in itself is a variable tissue composed of ciliated columnar cells, goblet cells, and in smaller bronchi occasional club cells. Subepithelial components of biopsies included cells such as leukocytes and fibroblasts, and extracellular matrix such as collagen and edema. Hence, cells other than epithelium contributed some RNA to analysis, which is a limit in this study. Nevertheless, assessment of in situ samples from a naturally occurring inducible model of asthma is previously unreported, and yielded unprecedented insight. Pre- and post-challenge bronchial biopsies from asthmatic and non-asthmatic horses were obtained, the transcriptome was derived from high throughput sequencing, and results were analyzed with a paired design to account for individual variation. Both groups of animals were of similar age, and as expected, the response to the inhaled challenge consisted of bronchoconstriction, marked airway neutrophilic inflammation, mucus accumulation and impaired airflow in asthmatic but not non-asthmatic animals.

RNA-seq data were analyzed using edgeR software. EdgeR and DESeq [49] are among the most widely used tools for RNA-seq analysis using raw counts. Both software tools use comparable methods with the exception of count normalization and dispersion estimation methods [50]. DESeq tends to be more conservative and edgeR more sensitive to outliers [51], but they yield overall highly similar results [51, 52]. Regardless of the method used, considering the genetic variability among horses, dispersion estimation would be more precise if data from a larger sample of individuals were analyzed. Therefore, within a limited sample size, the potential for false-positive results warrants caution for interpretation of individual findings.

Overall, the analysis identified 111 DE genes, which is a number similar to that identified in comparable studies in humans [53]. IHC analysis of representative bronchial biopsies confirmed that epithelial cell gene expression was representative of observed differential gene expression results. Transcriptome analysis of paired lung adenocarcinoma and non-neoplastic samples from non-smoking and smoking patients yielded 175 DE genes [53], while comparison of RNA-seq results of single bronchial biopsies from human asthmatics and healthy controls yielded 46 DE genes [53]. The latter study compared the bronchial transcriptome of asthmatics and non-asthmatics, rather than the difference in the response to an asthmatic challenge, as we did here. The workflow included different sequencing and statistical analyses, and the design did not account for inter-individual variability [54]. Nonetheless, similar to our results, up- and down-regulation of solute carrier (SLC) genes and up-regulation of an integrin-coding gene was detected. However, *B-cell lymphoma 2* (*BCL2*) expression was lower, and SCGB1A1 was higher in asthmatics compared to control, which contrasts with results in severe asthma in horses [17, 55] and may be due to different experimental design and different phases of disease being assessed.

Gene ontology and network analysis were subsequently used to characterize the DE genes. Since limited annotation of the horse genome constrains species-specific gene network and gene set analyses, human databases were utilized to expand interpretation of the findings. This approach yielded results consistent with prior association in asthma, but specific function of such gene products in horses remains to be ascertained. Finally, expression of the protein product of 4 DE genes was investigated immunohistochemically. Semi-quantitative assessment affirmed a link between gene and protein expression but factors such as RNA transcript stability and cell-to-cell variability in gene expression are incompletely accounted for with this approach. Hence, linked rather than individual genes should convey greater confidence for a role in asthma pathogenesis.

Several genes within overrepresented gene sets have been linked to asthma in humans*. S100A9* was the only gene shared across all gene sets, and is a calcium-binding protein highly conserved across species. S100A9 and related S100 proteins are highly expressed by neutrophils, and activate innate immune responses via interaction with TLR4 [56]. S100 proteins have also recently been identified to interact with airway epithelial cells to induce MUC5AC, the most abundant airway mucin [56]. Although *MUC5AC* was not differentially expressed in our study, it is known to be promoted by exposure to cytokines in both horses and humans in a concentration-and time-dependent manner [57, 58]. Our sampling time points may not have captured peak expression in all horses necessary reach significance in our stringent statistical analysis. Overexpression of MUC5A in horses with severe asthma [59] suggests a possible link of *S100A9* with mucus hyperproduction.

Network analysis of genes DE between asthmatic and non-asthmatic horses intimated MMP1, MMP9, TLR4 and IL8 as responsible for many interactions, and therefore to link and influence several asthmatic pathways and processes. *MMP9* was present in multiple significantly overrepresented GO gene sets. In addition, it had high BC and degree (number of direct connections) in the STRING network, meaning it accounted for many direct and indirect interactions within the network. *MMP9* is increased in human asthma [60], has anti-apoptotic effects in kidney injury and neutrophils [61, 62] and may be a link between inflammation and tissue remodeling [60, 63]. MMP9 also links serglycin (SRGN) to the main interaction cluster. *SRGN* is a proteoglycan that forms complexes with proMMP9 [64, 65] and is expressed in a variety of hematopoietic and non-hematopoietic cells [66]. Presence in immature granules has suggested a role in neutrophil differentiation [67], which could also influence neutrophil function in the context of granule release and cell death associated formation of neutrophil extracellular traps (NETs), also called NETosis.

Neutrophil infiltration was present in all asthmatic horses, consistent with the overrepresented neutrophil chemotaxis (GO:0030593) gene set. All genes within this gene set were previously associated with asthma pathogenesis except for *CSF3R*. CSF3 regulates production, differentiation and function of granulocytes, and overexpression is consistent with neutrophilic inflammation in equine severe asthma [68]. IL8 had among the highest BC and degree indicating a central role for linking components of the network. IL8 is a potent neutrophil attractant in the lung [69] and signals through CXCR1 and CXCR2 [70]. Increased *IL8* expression likely initiates and perpetuates neutrophil influx into the airways, but IHC also identified epithelial cells as a source of IL8. Secretion of IL8 by human epithelial cells can be promoted by exposure to TWEAK and activation of its receptor TNFRSF12A [71]. TWEAK is up-regulated in multiple tissues with inflammation, and associated with tissue changes such as remodeling [72]. Hence it may be plausible that epithelial cells up-regulate *TNFRSF12A* early in response to challenge, which in turn enhances IL8 production and maintains neutrophilic inflammation, leading to eventual proteolytic and oxidative injury.

It has been reported that asthmatic horses have dysregulated apoptosis of BAL but not peripheral blood leukocytes [73, 74]. Conversely, higher expression of *Immediate Early Response 3 gene (IER3)* identified in another study suggested dysregulated apoptosis in peripheral blood mononuclear cells of asthmatic horses [75]. Hence, the importance of leukocyte apoptosis in asthma of horses is unresolved. Significant overrepresentation of the apoptotic signaling pathway (GO:2,001,235) gene set was identified in tissue biopsies in this study, which included some extravasated leukocytes. This gene set included *S100A9, oncostatin M (OSM), THBS1, TNFRSF12A, stratifin (SFN), plasminogen activator urokinase receptor (PLAUR)* and *MMP9*. Other genes, such as *BIK*, a pro-apoptotic protein [76], had lower expression in asthmatic compared to non-asthmatic horses. BIK interacts with BCL2 and may protect airway mucous cells from apoptosis during remission from asthmatic exacerbation [55, 77]. Although *BCL2* was not DE, this may be a factor of the timing of biopsies and the lower proportion of mucous versus ciliated epithelial cells. Formation of neutrophil extracellular traps (NETs), another form of induced cell death, is prominent in BAL of horses with severe asthma [78]. The mechanism of NET formation is incompletely defined, but peptidyl arginine deiminase type IV (*PADI4)*, differentially expressed in asthmatic and non-asthmatic horses, contributes through citrullination of histones [79]. PADI4 expressed during NET formation may also promote coagulation through the release of serine proteases [80]. The positive regulation of blood coagulation (GO:0030194) gene set was overrepresented and included *S100A9, PLEK, THBS1* and *TLR4*. Chronic up-regulation of coagulation [81] and systemic inflammation were reported in horses with severe asthma [82], and activation of the coagulation cascade [81] together with impaired epithelial repair [83] are features of human asthma. Hence, several lines of evidence suggest concurrence of hemostatic, coagulative and tissue repair processes with neutrophil activation in severe asthma. Furthermore, apoptosis and NETosis appear to be component of asthma as suggested by differential expression and linkage of genes in these pathways.

Among the DE genes are several of potential interest that are not part of overrepresented gene sets or interaction networks. Differences in cell cycle-related gene expression in peripheral blood mononuclear cells (PBMCs) have been reported in asthmatic horses [75]. CDC25A, a cell cycle-related gene, was not part of any network, but was highly DE and likely influences cell cycle and differentiation in bronchial epithelium during inflammation, as it does in other contexts such as neoplasia [84, 85]. Genes such as *ENSECAG00000014899* and *ENSECAG00000017229* (potential orthologs of human *KRT6* genes), *KRT17* and *ENSECAG00000007450* (potential ortholog of human *SPRR1A/B*) and *transglutaminase 1* (*TGM1*) were not identified in network interactions, but are likely to function in squamous metaplasia [86]. Recent reports implicate hedgehog (HH) pathway-associated molecules in lung disease of humans [87]. Single nucleotide polymorphisms (SNPs) in *Patched-1 (PTCH1),* a DE gene*,* and *hedgehog-interacting protein (HHIP)*, involved in the hedgehog pathway, have been associated with lung function in humans [88, 89]. In conjunction, *PTCH1*, *HHIP* and family with sequence similarity 13, member A (*FAM13A*) predicted lung function abnormalities in an asthmatic cohort [88].

Five differentially expressed genes linked to regulation of the circadian clock were consistently down-regulated in asthmatic animals: *CIART (CHRONO), PER3, DBP, TEF, ADRB2* and *NR1D2*. CIART is part of a transcriptional repressor of the mammalian clock, and contributes to a suppressive glucocorticoid response that is dependent on physiological stress [90]. PER3 is expressed in a circadian pattern in the brain suprachiasmatic nucleus and also in peripheral tissues [91]. Changes in this group of genes may indicate disrupted circadian rhythm in the asthmatic lung. *NR1D2* and *PER3* have been associated with asthma in mice through bioinformatics analysis of genes and pathways [92]. *ADRB2* has been directly linked to circadian leukocyte recruitment [93]. In addition, in mice club cells may have a role in the circadian regulation of the lung through rhythmic CXCL5 (orthologue gene to CXCL6 in horses and humans) responses and loss of this regulation leads to aberrant neutrophil influx [94]. SCGB1A1 is considered a key molecule for homeostasis in the lung, and club cells and SCGB1A1 are reduced in horses with severe asthma [78]. Club cell depletion may result from impaired epithelial precursor cell recruitment and differentiation, and trigger further dysregulated pulmonary circadian rhythm.

## Conclusions

There were pronounced differences in the epithelial response to challenge in asthmatic and non-asthmatic horses. Genes identified include many with prior association in asthma, and novel genes that potentially link pathogenic mechanisms. For candidate genes of interest, further functional characterization should be undertaken. For example, a protein-protein interaction assay in BALF using recombinant versions of protein of interest might be informative. In addition, investigation of epigenetic markers may further characterize environmental influences on genes.

## Additional files

Additional file 1: Table S1. Clinical parameters, bronchoalveolar lavage and pulmonary function results. **Table S2.** Top genes differentially expressed between horses with and without asthma after challenge, ranked by logFC. (XLSX 31 kb)

Additional file 2: Figure S1. Immunoblots assessing antibody reactivity for (A) TNFRSF12A, (B) PTCH1, (C) CDC25A and (D) IL8. Only antibodies yielding a single band of expected size were used in subsequent immunohistochemical assays. (TIFF 69 kb)

## Abbreviations

BAL: Bronchoalveolar lavage
BC: Betweenness centrality
BCL: B-cell lymphoma
BCV: Biological coefficient of variance
CDC25A: Cell division cycle 25 homolog A
CXCR: X-C chemokine receptor
DE: Differentially expressed
GO: Gene ontology
GOBP: GO overrepresentation for biological processes
IHC: Immunohistochemistry
IL: Interleukin
KRT: Keratin
MDS: Multidimensional scaling
MMP: Matrix metalloproteinase
PANTHER: Protein ANalysis THrough Evolutionary Relationships
PFT: Pulmonary function testing
PRR: Pattern recognition receptor
STRING: Search Tool for the Retrieval of Interacting Genes/Proteins
TLR: Toll-like receptor
TNFRSF: Tumor necrosis factor receptor superfamily
TWEAKR: Tumor necrosis factor-like weak inducer of apoptosis receptor
