# Supplementary figures and images for: Impaired response of the bronchial epithelium to inflammation characterizes severe equine asthma
Source: BMC Genomics. 2017 Sep 8;18:708. doi: 10.1186/s12864-017-4107-6 (PMC5591550; doi:10.1186/s12864-017-4107-6)

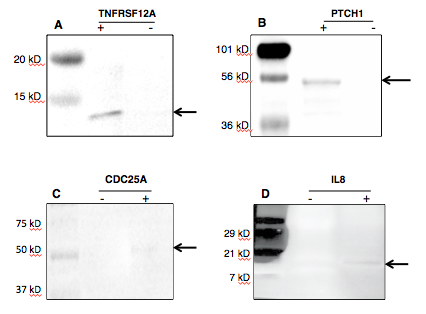

Supplement: Supplementary file 2 — Immunoblots assessing antibody reactivity for (A) TNFRSF12A, (B) PTCH1, (C) CDC25A and (D) IL8. Only antibodies yielding a single band of expected size were used in subsequent immunohistochemical assays. (TIFF 69 kb) [file 12864_2017_4107_MOESM2_ESM.tiff]
